# Unlocking Success in Counseling: How Personality Traits Moderates Its Effectiveness

**DOI:** 10.3390/ejihpe14100174

**Published:** 2024-09-24

**Authors:** Alexandro Fortunato, Silvia Andreassi, Costanza Franchini, Gaetano Maria Sciabica, Mara Morelli, Antonio Chirumbolo, Anna Maria Speranza

**Affiliations:** 1 Department of Dynamic, Clinical Psychology and Health, Faculty of Medicine and Psychology, Sapienza University of Rome, 00185 Roma, Italy; silvia.andreassi@uniroma1.it (S.A.); costanza.franchini@uniroma1.it (C.F.); gaetanomaria.sciabica@uniroma1.it (G.M.S.); mara.morelli@uniroma1.it (M.M.); annamaria.speranza@uniroma1.it (A.M.S.); 2 Department of Psychology, Faculty of Medicine and Psychology, Sapienza University of Rome, 00185 Roma, Italy; antonio.chirumbolo@uniroma1.it

**Keywords:** psychological counseling, university counseling services, university counseling effectiveness, personality, depression, emerging adulthood, university students

## Abstract

Psychological distress is widespread among university students, with depression being notably more prevalent compared to the general population. University counseling services are crucial for addressing these mental health challenges, and numerous studies have demonstrated their effectiveness in reducing psychological distress and improving overall well-being. However, there is limited research on what factors predict the success of university counseling. This study aims to evaluate whether counseling improves well-being, specifically by reducing depressive symptoms, and to explore whether personality traits influence counseling outcomes. Participants included 125 Italian university students (64.8% female, mean age = 22.69; SD = 3.04) who utilized counseling services. They completed a socio-demographic questionnaire, the Beck Depression Inventory-II (BDI-II), and the Personality Inventory for DSM-5-TR (PID-5-TR) at three points: immediately after the intake interview (T0), just before the intervention (T1), and after the fourth session (T2). Linear mixed models were used to analyze changes in depression levels, revealing a significant reduction in depressive symptoms from pre- to post-intervention. Among personality traits, only antagonism showed a significant interaction with time. Additionally, higher detachment scores were associated with higher depression levels. These findings emphasize the need for focused attention on students’ emotional issues and suggest that personality traits may influence the effectiveness of counseling.

## 1. Introduction

The mental well-being of university students is receiving more focus, resources, and scientific investigation [1]. This heightened interest stems from its connection to substantial social and psychological problems. Research indicates that psychological distress is prevalent among university students, with notable concerns including anxiety, depression, interpersonal difficulties, and suicidal thoughts [2,3,4,5,6,7,8]. Specifically, depression and sadness are more prevalent among university students worldwide compared to the general population [5,9,10,11,12,13]. This is why depressive symptoms are of particular interest to university counseling services and can serve as an effective measure of outcomes.

The causes of this distress can be traced to the new challenges faced during the “emerging adulthood” stage of development [14,15,16], especially for university students who must manage these challenges while also performing academically. This, combined with a growing awareness of mental health importance and reduced stigma [5,6], has led to increased demand for psychological support from university students worldwide [17].

To meet this growing demand, university counseling services are essential in addressing the escalating mental health needs of students [18,19,20]. Numerous studies, both Italian and international, have shown the effectiveness of these services in alleviating psychological distress and enhancing the overall well-being of university students [4,21,22,23,24,25].

Despite the evidence of effectiveness, few studies have examined factors that might predict the success of university counseling services. Predictive factors can be divided into state variables, such as anxiety and depression, where lower initial levels are intuitively linked to a higher likelihood of successfully completing the intervention [24,26]. Conversely, trait variables, being less influenced by the current situation, can provide more insight into which students might benefit the most from counseling. Trait variables refer to stable and persistent characteristics of an individual’s personality. These traits are considered relatively constant over time and across different situations, and they influence behavior, emotions, and thoughts. In this context, personality is a key variable that could best explain the outcome of counseling. Due to its predictive value, it is one of the most extensively studied variables in relation to the outcomes of psychotherapeutic treatment, hospital treatment, and similar interventions [27].

Personality traits, as defined by the Five Factor Model (FFM), offer a comprehensive framework for investigating human personality and have been widely used in numerous research studies, particularly to construct and validate assessment tools [28]. The FFM posits that five broad domains—neuroticism (or emotional instability vs. stability), extraversion (vs. introversion), openness (or unconventionality), agreeableness (vs. antagonism), and conscientiousness (or constraint vs. disinhibition)—encompass the majority of individual differences in personality. Each of these dimensions can be associated with various aspects of psychological treatment, and for this reason, they can be used to effectively capture the dynamics of outcome prediction.

The Diagnostic and Statistical Manual of Mental Disorders, Fifth Edition, Text Revision (DSM-5-TR; [29]), presents a new approach for evaluating personality traits, especially in clinical settings, referred to as the Alternative Model for Personality Disorders (AMPD). This approach seeks to offer a clearer framework for diagnosing and treating personality disorders, with the AMPD domains representing maladaptive extremes of the FFM.

The AMPD [29] includes five main domains of pathological personality traits:

- Negative Affectivity: this domain closely aligns with neuroticism in the FFM. It encompasses traits related to frequent and intense experiences of various negative emotions, such as anxiety, depression, and emotional instability, along with their behavioral and interpersonal effects.
- Detachment: This domain mirrors the low end of extraversion in the FFM. It includes traits like avoidance of socioemotional interactions, ranging from casual encounters to deeper relationships, and a limited range of emotional experiences, particularly a reduced ability to experience pleasure.
- Antagonism: This domain somewhat parallels low agreeableness in the FFM. It covers traits such as manipulativeness, grandiosity, and hostility. Individuals with high levels of antagonism tend to have an inflated sense of self-importance and expect special treatment, often disregarding others’ needs and using people for personal benefit.
- Disinhibition: This domain is partly akin to low conscientiousness in the FFM. It involves traits such as impulsivity, irresponsibility, and a lack of planning. It is characterized by a focus on immediate gratification, resulting in behavior driven by current emotions and external stimuli without consideration of past experiences or future consequences.
- Psychoticism: This domain does not have a direct counterpart in the FFM. It represents a departure from rationality and includes traits like unusual thinking, eccentricity, and atypical perceptions. It involves displaying a wide range of culturally incongruent behaviors and cognitions, affecting both cognitive processes (such as perception and dissociation) and content (such as beliefs).

This alternative model was developed to improve the understanding and diagnosis of personality disorders, emphasizing the pathological dimensions that can disrupt daily life, patient well-being, and treatment effectiveness. The AMPD of the DSM-5-TR [29] has been adapted into a diagnostic tool known as the Personality Inventory for DSM-5 (PID-5; [30]), which is designed to evaluate pathological personality traits. The Personality Inventory for DSM-5 Brief Form (PID-5-BF; [31]) is a condensed version of the PID-5, intended to serve as a rapid screening instrument. This brief form preserves the core five domains but features fewer items per domain, making it quicker and simpler to use. The PID-5-BF is used in clinical settings to quickly evaluate pathological personality traits in patients.

The interplay between personality traits and the effectiveness of psychological treatment has become a major focus of recent research. Investigating how innate personality features affect therapeutic processes and outcomes can provide valuable insights for diagnosing, improving treatment effectiveness, and customizing therapeutic strategies. Moreover, the bidirectional nature of the relationship between personality and psychological treatment is noteworthy. Not only can personality traits impact treatment results, but the therapy itself may also bring about changes in an individual’s personality. This interactive dynamic highlights the need to view personality not just as a fixed predictor but as a flexible element within the therapeutic setting.

Despite substantial evidence supporting these associations, there are still gaps in fully understanding how personality traits affect treatment outcomes. Results are not always clear-cut and can vary depending on the type of treatment and its duration. Moreover, these studies often do not account for the hierarchical structure of the data, focusing more on the direct effects of treatment adherence than on the moderating effects of personality traits on treatment outcomes. Additionally, control samples or patients on waiting lists are frequently lacking.

In the research conducted by Rek et al. [32], maladaptive traits assessed with the PID-5-BF did not predict changes in depressive symptoms following cognitive-behavioral therapy (CBT) or schema therapy (ST). As a result, these traits did not moderate the effectiveness of the treatments. Nevertheless, the maladaptive trait domains did show a reduction throughout the course of treatment, reflecting an overall improvement. Similarly, the investigation by Osma et al. [33] revealed that initial personality scores did not predict the changes observed during CBT for depression and anxiety symptoms, though negative traits tended to improve. Conversely, Rodriguez-Seijas et al. [34] found a connection between maladaptive personality traits, as assessed by the PID-5-BF, and the premature discontinuation of hospital treatments. Participants who terminated treatment early exhibited higher levels in all PID-5-BF domains, except antagonism, compared to those who completed their treatment. Additionally, increases in the disinhibition and psychoticism domains were associated with about double the likelihood of early discontinuation compared to normative levels.

Two systematic literature reviews presented interesting findings. Molloy et al. [35] demonstrated a positive correlation between the conscientiousness trait from the FFM and treatment adherence. Meanwhile, Bucher et al. [27] explored various personality traits’ correlations with intervention outcomes, revealing numerous specific relationships. In terms of the FFM, the study indicated that lower levels of neuroticism and higher levels of extraversion, agreeableness, conscientiousness, and openness are linked to more favorable outcomes. Specifically, agreeableness was positively associated with the therapeutic alliance. Personality traits were also related to various outcomes in different ways, depending on moderators. For example, treatment duration moderated the links between traits and outcomes, suggesting that these effects are amplified by longer interventions.

Similar contradictory findings arise when examining personality disorders (PDs) rather than traits. Mulder [36], in line with review by Bucher et al. [27], observed that elevated neuroticism scores often predict worse treatment outcomes for depression, particularly in long-term follow-ups. However, overall, the study indicates that personality disorders do not consistently predict whether an intervention will result in better or worse outcomes.

Even when examining counseling, the limited research available presents mixed findings regarding the predictive power of personality on intervention outcomes. Speranza et al. [24] found that personality traits assessed using the PID-5-BF did not seem to predict the results of the intervention. Instead, state variables were shown to play a significant role in enhancing psychological well-being following the counseling sessions. In contrast, Biasi et al. [16], who employed the Minnesota Multiphasic Personality Inventory-2 (MMPI-2; [37]), discovered that the dimensions of the inventory were useful in predicting counseling outcomes. Notably, the psychopathic deviate dimension was effective in predicting a greater benefit from counseling.

Given the lack of definitive answers in the literature and the importance of understanding how counseling functions and by which variables it is affected, we sought to determine if a more advanced prediction model could provide clearer insights into personality prediction. Using a linear mixed model and incorporating waiting list data along with pre- and post-intervention data, our goal was to identify which personality traits benefit most from counseling interventions and which do not. This understanding would aid in optimizing counseling services and customizing interventions to better meet students’ needs.

Based on these considerations, this study has two primary research aims: first, to determine whether counseling is effective in enhancing well-being, specifically in reducing depressive symptoms; and second, to examine whether personality traits influence the outcome of the counseling intervention.

The following hypotheses will be tested:

**Hypothesis** **1:** *That depressive symptoms in students, as measured by the Beck Depression Inventory-II (BDI-II; [38]), will decrease after the counseling intervention, according to the literature [24,39].*

**Hypothesis** **2:** *That personality traits, assessed at T0 using the PID-5-BF, will moderate the decrease in the depressive symptoms, as measured by BDI-II over time (T0 and T1 versus T2). Based on the literature, we expect that low levels of negative affectivity, detachment, antagonism, disinhibition, and psychoticism will moderate the decrease in depressive symptoms over time [27,37].*

## 2. Materials and Methods

### 2.1. Participants and Procedure

The study participants were students who attended counseling at the Counseling Center of Sapienza University of Rome between May 2023 and May 2024. During this period, 290 university students were referred to the counseling intervention and filled the first completion after the brief initial interview. Of these, 125 students (43%) filled all three completions required to assess the progress of the intervention (two pre-test completions and one post-test completion), while 23% (*n* = 67) of students dropped out of the intervention after the initial interview, and 34% (*n* = 98) of students completed the intervention but did not fill out the post-test questionnaires.

Thus, the final sample involved 125 university students who had completed the four-session counseling program at the Counselling Center of Sapienza University. To verify whether the sample size used in the study was adequate for detecting significant effects, a post-hoc power analysis was conducted using G*Power 3.1.9.7 [40]. With a significance level of α = 0.05, a small effect size (f-square = 0.15), and a sample size of 125 subjects, the analysis indicated a power of 0.96, which is well above the 0.80 level commonly accepted as a good statistical power [41]. This high-power value suggests a very robust likelihood of detecting a significant effect, thereby providing strong assurance that the results are reliable and the risk of a Type II error is minimized.

This program consists of four weekly sessions followed by a follow-up session 3 months later. Designed with a psychodynamic approach, the counseling aims to clarify and address the emotional experiences of students, assisting them in resuming their developmental progress if it has been interrupted or hindered [14,42]. 

Before starting, participants filled out an online informed consent form and provided their biographical details on an intake form. They then had a brief initial interview with a psychologist to assess their needs and recommend the most appropriate treatment. Participants were excluded if they (a) had a serious psychiatric condition (such as psychotic disorders or bipolar disorder) or (b) were already undergoing psychotherapy or other psychological treatments. Those with severe psychiatric conditions identified during the intake were referred to specialized services. Assessment tools were administered through the Qualtrics platform, with alphanumeric codes ensuring participant confidentiality. Questionnaires were completed at three time points: immediately after the intake interview (T0); just before the intervention began (T1); and at the conclusion of the intervention (after the fourth session) (T2). The intervention lasted about a month, which was the same duration as the waiting period between T0 and T1. Comparing the waiting period (T0–T1) with the intervention period (T1–T2) allows for the evaluation of changes attributable to the counseling. The counseling sessions were led by 29 clinicians from our university’s post-graduate psychotherapy training program, who had received specific training for this counseling approach.

The present study was approved by the ethics committee of Sapienza, University of Rome (number protocol: 96/2023) and conducted in accordance with the Ethical Principles for Medical Research Involving Human Subjects (Declaration of Helsinki).

### 2.2. Measures

Socio-demographic questionnaire: A customized questionnaire was created by the counseling service to gather the following demographic details: gender, age, academic courses, degree program and reasons for accessing counseling.

Beck Depression Inventory-II (BDI-II; [38]): The BDI-II is a self-report tool designed to evaluate the intensity of depressive symptoms experienced by adolescents and adults over the past 2 weeks. It features 21 items rated on a 4-point Likert scale (from 0 = absence of symptoms, to 3 = strong presence of symptoms), covering two dimensions: somatic-affective symptoms (12 items, e.g., loss of pleasure, loss of energy, fatigue) and cognitive symptoms (9 items, e.g., self-esteem, self-criticism, sense of worthlessness). A cut-off of 31 was used to determine severe depression. In this study, Cronbach’s α values were as follows: 0.87 at T0, 0.93 at T1, 0.92 at T2.

Personality Inventory for DSM-5-Brief Form (PID-5-BF; [31]): The PID-5-BF is a self-report tool designed to evaluate five personality trait domains: negative affectivity, detachment, antagonism, disinhibition, and psychoticism. It was created by selecting 25 items from the original PID-5′s 220 items [30]. Each domain is represented by five items, which are rated on a 4-point scale (from 0 to 3), with higher scores indicating more pronounced dysfunction. Fossati et al. [43] demonstrated that the instrument has strong internal consistency and test-retest reliability in a sample of Italian adolescents. In this study, the Cronbach’s α values of personality traits assessed at T0 were: 0.70 for negative affectivity, 0.86 for detachment, 0.82 for antagonism, 0.82 for disinhibition, 0.81 for psychoticism.

### 2.3. Statistical Analyses

All the analyses were conducted with the software Jamovi version 2.4.11 [44]. We firstly performed descriptive statistics to examine the characteristics of participants. 

To address our first aim (i.e., analyzing the differences, for each student, in depression scores between T0, T1, and T2), we performed a linear mixed model (LMM). We used this model since it allows us to evaluate the dependent variable while considering differences in within-participants and within-therapists measurements. Since our model involved multiple observations for each subject as well as different therapists, we considered the participants and the therapists as cluster variables. Participants’ age (measured in years) and gender (coded as 0 = female, 1 = male) were considered as covariates, while the time of completion (T0, T1, T2) as a factor variable.

To address our second aim (i.e., analyzing the role of personality traits on outcome intervention), we performed five linear mixed models, one for each personality trait assessed at T0. In this case, each personality trait was entered as an independent variable and used as a moderator of the relationship between time (T0, T1, T2) and depression scores, controlling for participants’ gender and age and considering participants and therapists as cluster variables.

The significance level was set at *p* < 0.05. To reduce the risk of a type I error, post-hoc Bonferroni tests were conducted for the “time” variable. 

## 3. Results

We initially performed descriptive statistics for the sample (see Table 1). Our sample showed a prevalence of female students (*n* = 81, 64.8%), mainly involved in bachelor’s degrees (consistent with the mean age). This gender distribution is consistent with the data reported by Sapienza University regarding the percentage of female and male students enrolled in university courses. The division of faculties adheres to the structure of the faculties at Sapienza. Students primarily seek counseling for emotional and relational issues, with academic or other difficulties being much less common reasons.

Table 2 presents the means and standard deviations of the variables examined at various time points.

Concerning the first hypothesis, we conducted a linear mixed model in order to evaluate the decrease in depression levels measured with the BDI-II between the pre-intervention (T0 and T1) and post-intervention (T2) scores. The linear mixed model conducted on the BDI-II scores showed that the participants reported no significant difference in the depression levels between the measures taken pre-intervention (T1–T0; *p* = 0.721) while they reported a significant decrease between the pre-intervention measure and the post-intervention measure (T2–T0; *p* < 0.001). For the linear mixed-effects model, fixed effects explained 8.4% of the variance in depression scores (*R*^2^ Marginal = 0.084), whereas the entire model (comprising random effects) explained 75.7% of the variance (*R*^2^ Conditional = 0.757). The Intraclass Correlation Coefficient (ICC) values of the cluster variables, were 0.107 for “therapists” and 0.726 for “students”, showing that the 25.41% of the variance of the change in BDI-II scores between pre- and post-intervention is explained by taking into account the multi-level data (see Table 3). Subsequent post-hoc analyses showed that after the counseling intervention (T2), participants showed a significant decrease in depression levels (*p* < 0.001) compared to both pre-intervention measures (T0 and T1).

To test the second hypothesis, we conducted five sets of linear mixed models, for each personality trait assessed by PID-5-BF (i.e., negative affectivity, detachment, antagonism, disinhibition, psychoticism) on depression outcomes assessed by BDI-II. As shown by the linear mixed model, depressive symptoms decrease significantly between T1 and T2 for all students. The decrease is not significant between T0 and T1 while students are waiting for the intervention to begin.

Regarding negative affectivity, the LMMs’ results highlighted that it did not moderate the relationship between time and depression (see Table 4). 

Regarding detachment, the LMMs’ results highlighted that it did not moderate the relationship between time and depression. However, when considering the effect of the single variable on depression, higher scores of detachment led to us significantly predicting depression over time (*p* = 0.043) (see Table 5).

**Figure 1:**
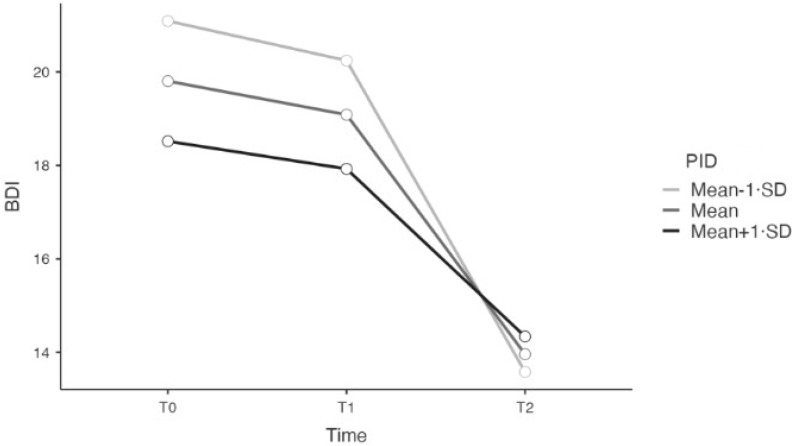
The significant interaction effect of antagonism.

Regarding antagonism, the LMMs’ results showed a significant interaction effect between time and depression (*p* = 0.012). See Table 6 and Figure 1. The relationship between time and depression was significant and negative for each level of antagonism (Mean −1 SD; Mean; Mean +1 SD). However, the interaction effect from T0 to T2 was stronger for level Mean −1 SD (B = −7.510; SE = 0.931; t = −8.064).

Regarding disinhibition, the LMMs’ results highlighted that it did not moderate the relation between time and depression (see Table 7). 

Regarding psychoticism, the LMMs’ results highlighted that it did not moderate the relationship between time and depression (see Table 8).

## 4. Discussion

The present study aims to evaluate the effectiveness of a counseling intervention and the influence of personality traits on this effectiveness. To address the first aim, we examined whether counseling is effective in reducing depressive symptoms. For the second aim, we investigated whether personality traits can moderate the reduction in depressive symptoms. We focused on depressive symptoms because of their prevalence among university students and the recommendation by Vescovelli et al. [45] that university counseling services address affective disorders specifically. This aligns with the fact that counseling is most often sought for emotional and relational issues, with academic or other difficulties being far less common reasons. Moreover, understanding the factors that influence the effectiveness of university counseling is crucial for designing interventions that meet students’ needs [46]. Our findings provide further evidence of the counseling intervention’s effectiveness in enhancing well-being; this is consistent with previous research [18,24,39].

The sample comprised 125 students, predominantly female, which is consistent with previous studies on counseling [4,24,45] and reflects the gender distribution at our university. Furthermore, we can suggest that the predominance of female students may be due to women being more likely than men to seek psychological support [47,48].

To test the first hypothesis, we used an LMM to analyze changes in depression levels, measured by the BDI-II, between pre-intervention (T0 and T1) and post-intervention (T2) scores. This model accounted for repeated measurements, the hierarchical structure of the data (students nested within therapists) and controlled for age and gender. Results showed no significant difference in depression levels between the pre-intervention measurements. However, there was a significant decrease in depression levels between pre- and post-intervention measures, suggesting that the decrease in depression is attributable to the counseling intervention and that these data are consistent with the literature [18,40,49]. No differences emerged regarding age and gender, indicating that all participants benefited equally from the intervention.

These data not only confirm the effectiveness of counseling in reducing symptoms but also highlight the specific areas in which counseling is particularly impactful. For instance, it can be hypothesized that the reduction in depressive symptoms is linked to a renewed sense of confidence in the future, which may be one of the core components of university counseling [24]. Students in the emerging adulthood phase often experience feelings of disorientation, fear, and uncertainty about the future. Counseling plays a crucial role in addressing these challenges: it can reinvigorate stalled developmental processes, help students reassess their resources and strengths, refocus on their desires, and regain a more complete sense of self [14].

For the second hypothesis, we performed five LMMs, one for each personality trait measured by the PID-5-TR (negative affectivity, detachment, antagonism, disinhibition, psychoticism), to analyze their role as moderators in the relationship between time and depression scores. We controlled for gender and age and considered the hierarchical structure of the data.

Results showed that negative affectivity, detachment, disinhibition, and psychoticism did not moderate the relationship between time and depression, although counseling was effective for all maladaptive personality traits. Interestingly, only antagonism showed a significant interaction effect, with lower levels of antagonism associated with a more pronounced decrease in depressive symptoms. Our findings are consistent with expectations from previous literature [27,35,36], suggesting that low levels of maladaptive traits moderate the outcome. Specifically, the role of low antagonism as a moderator aligns with the studies by Rodriguez-Seijas et al. [34] and Biasi et al. [16]. Although the first study, which examines the direct relationship between antagonism and discontinuation of hospital treatment (rather than its role as a moderator), shows only partial alignment with our findings, it still provides a relevant context. The second study, which found that the psychopathic deviate dimension of the MMPI-2 was effective in predicting greater benefit from counseling, supports our results given that antagonism is a component of antisocial and psychopathic personality traits.

These data do not offer a definitive conclusion but provide new insights into these aspects. It is noteworthy that the same personality trait identified in this study was also found in Biasi et al. [16]. This is important because traits such as transgression, impulsivity, and risk-seeking are critical to monitor during emerging adulthood. Additionally, in brief interventions like university counselling, there is limited time to build a strong counsellor-student relationship. As a result, the more predisposed the student is to engaging in the relationship and showing less antagonism, the more effective the intervention is likely to be.

Counseling provides students with a private and safe space to express themselves freely, which aids mentalization processes [50]. This allows students to reassess their functioning and develop curiosity about their feelings and experiences. While counseling sessions may not provide enough time to fully explore mental functioning, the observed improvement in maladaptive personality traits suggests that a change mechanism is set in motion, potentially continuing even after the intervention. This process of change, reflection, and reorganization [22] appears to be more easily facilitated in students with lower levels of antagonism. Future studies should aim to confirm whether these findings hold true in follow-up assessments. Additionally, further research is needed on the influence of personality traits evaluated with different assessment tools (i.e., clinician-report questionnaires) on counseling outcomes.

Additionally, detachment emerged as a predictor of depression, with higher detachment scores associated with higher depression scores. This is consistent with the DSM-5-TR description of this trait [29]. One might assume that high negative affectivity, characterized by a range of negative emotional aspects including depression [29], would also predict high levels of depression. However, negative affectivity not only encompasses negative emotions but also signifies a general flattening of responses to negative emotions, leading to emotional instability.

In conclusion, it is important to highlight both the strengths and limitations of this study. It underscores the importance of addressing students’ emotional problems, particularly depression, through university counseling [24,45]. The use of an LMM to handle repeated measures and variations among counselors strengthens the reliability of the results. Repeated measures at three time points allow for a detailed examination of changes in depressive symptoms over time. The inclusion of a waitlist control and control for variability among counselors further supports the attribution of results to the counseling intervention. Despite some limitations, such as the referral of students with severe psychiatric conditions to other services and the exclusive use of self-report tools, these findings are promising. Furthermore, the sample consists of university students from a single institution who actively sought help. This means that the data may not be generalizable to the broader population.

Finally, it is important to note that the study only includes students who have completed the intervention. Therefore, future research should explore the personality traits of those who did not complete it to understand how to better retain participants in treatment.

## 5. Conclusions

In summary, this study demonstrates the effectiveness of a university counseling intervention in reducing depressive symptoms, with robust statistical analysis using a linear mixed model. The findings regarding antagonism provide valuable insights into how personality traits might influence the effectiveness of counseling, though further research is warranted to deepen the understanding of these relationships and to explore the impact of personality traits on the effectiveness of counseling interventions. If this association is confirmed in future studies, it would be important to reflect on the counseling approach for students with high levels of antagonism. These students should be provided, as much as possible, with an environment that promotes mentalization and relational processes, while fostering their ability to reflect on their own behavior and its consequences.

## Figures and Tables

**Table 1:** Descriptive sample statistics.

	*N* = 125 % (*n*)
Gender	64.8% F (81)
Mean age	22.69 (±3.04)
University courses	
Architecture	0.8% (1)
Economics	5.6% (7)
Pharmacy and medicine	10.4% (13)
Law	5.6% (7)
Civil and industrial engineering	2.4% (3)
Information engineering, computer science and statistics	8% (10)
Literature and philosophy	20.8% (26)
Medicine and dentistry	8.8% (11)
Medicine and psychology	7.2% (9)
Mathematical, physical and natural sciences	17.6% (22)
Political sciences, sociology and communication	7.2% (9)
Mechanical and aerospace engineering	5.6% (7)
Degree program	
Bachelor’s degree	48.8% (61)
Master’s degree	28% (35)
Single cycle	19.2% (24)
PhD course	4% (5)
Reasons for accessing counseling	
Emotional/relational-psychological difficulties	64.8% (81)
Difficulties related to study and university career	24% (30)
Other issues	11.2% (14)
BDI scores above the cut-off at pre-test	11.2% (14)
BDI scores above the cut-off at post-test	8% (10)

**Table 2:** Means and standard deviations of the main variables.

	T0	T1	T2
Variable	M (SD)	M (SD)	M (SD)
Depression	20.04 (9.78)	19.37 (10.02)	14.35 (9.26)
Negative Affectivity	1.97 (0.73)		
Detachment	1.39 (0.80)		
Antagonism	0.87 (0.75)		
Disinhibition	1.13 (0.75)		
Psychoticism	1.39 (0.77)		

**Table 3:** Linear mixed model: change in depression scores (BDI-II) over time.

				Confidence Intervals				
Names	Effect	Estimate	SE	Lower	Upper	df	t	*p*
Fixed coefficients								
Intercept		17.548	0.910	15.758	19.338	16.7	19.282	<0.001
T1	T1–T0	−0.775	0.659	−2.071	0.520	234.6	−1.177	0.240
T2	T2–T0	−5.791	0.657	−7.082	−4.499	234.4	−8.818	<0.001
Gender		−2.898	1.693	−6.228	0.431	111.6	−1.712	0.090
Age		−0.145	0.266	−0.668	0.379	110.0	−0.543	0.588
**Random Components**					**Variance**	**SD**	**ICC**	
Participants’ intercept					67.19	8.20	0.726	
Therapists’ intercept					3.04	1.74	0.107	
Residual					25.41	5.04		
**Model Fit**					**R^2^**	**df**	**LRT X^2^**	***p***
Conditional					0.757	4	234.522	<0.001
Marginal					0.084	4	81.934	<0.001
**Post-Hoc Comparison: Time**				**Difference**	**SE**	**df**	**t**	***p***
T0 vs. T1				0.755	0.659	235	1.18	0.721
T0 vs. T2				5.791	0.657	234	8.83	<0.001
T1 vs. T2				5.015	0.653	234	7.68	<0.001

Note. Gender was coded as follows: 0 = female; 1 = male; SE = standardized error; df = degrees of freedom; SD = standard deviation; ICC = intraclass coefficient.

**Table 4:** Linear mixed-effects model with negative affectivity as moderator.

				Confidence Intervals				
Names	Effect	Estimate	SE	Lower	Upper	df	t	*p*
Fixed coefficients								
Intercept		17.672	0.854	15.993	19.352	112	20.700	<0.001
T1	T1–T0	−0.716	0.671	−2.037	0.604	227	−1.067	0.287
T2	T2–T0	−5.845	0.669	−7.161	−4.529	227	−8.735	<0.001
Gender		−2.456	1.738	−5.875	0.963	112	−1.413	0.160
Age		−0.113	0.266	−0.637	0.411	112	−0.425	0.671
NA		1.908	1.167	−0.387	4.203	112	1.636	0.105
T1 × NA	T1–T0	−0.339	0.932	−2.173	1.495	227	−0.363	0.717
T2 × NA	T2–T0	−0.288	0.932	−2.121	1.545	227	−0.310	0.757
**Random Components**					**Variance**	**SD**	**ICC**	
Participants’ intercepts					68.40	8.27	0.725	
Therapists’ intercepts					0.816	0.903	0.031	
Residual					25.97	5.10		
**Model Fit**					**R^2^**	**df**	**LRT X^2^**	***p***
Conditional					0.755	9	232.293	<0.001
Marginal					0.104	7	84.060	<0.001
**Post-Hoc Comparison: Time**				**Difference**	**SE**	**df**	**t**	***p***
T0 vs. T1				0.717	0.671	227	1.07	0.860
T0 vs. T2				5.845	0.669	227	8.74	<0.001
T1 vs. T2				5.128	0.671	227	7.64	<0.001

Note. Gender was coded as follows: 0 = female; 1 = male; SE = standardized error; df = degrees of freedom; SD = standard deviation; ICC = intraclass coefficient; NA = negative affectivity.

**Table 5:** Linear mixed-effects model with detachment as moderator.

				Confidence Intervals				
Names	Effect	Estimate	SE	Lower	Upper	df	t	*p*
Fixed coefficients								
Intercept		17.671	0.847	15.9223	19.419	14.9	19.879	<0.001
T1	T1–T0	−0.715	0.667	−2.0265	0.597	227.2	−1.072	0.285
T2	T2–T0	−5.845	0.665	−7.1524	−4.537	227	−8.793	<0.001
Gender		−2.571	1.706	−5.9273	0.785	109.0	−1.507	0.135
Age		−0.159	0.265	−0.6803	0.361	102.7	−0.602	0.549
Det		2.144	1.048	0.0829	4.205	111.6	2.046	0.043
T1 × Det	T1–T0	−0.984	0.849	−2.6541	0.686	227.0	−1.159	0.248
T2 × Det	T2–T0	0.499	0.849	−1.1708	2.169	227	0.588	0.557
**Random Components**					**Variance**	**SD**	**ICC**	
Participants’ intercepts					66.34	8.15	0.721	
Therapists’ intercepts					2.02	1.42	0.073	
Residual					25.63	5.06		
**Model Fit**					**R^2^**	**df**	**LRT X^2^**	***p***
Conditional					0.759	9	236.895	<0.001
Marginal					0.116	7	88.662	<0.001
**Post-Hoc Comparison: Time**				**Difference**	**SE**	**df**	**t**	***p***
T0 vs. T1				0.715	0.667	227	1.07	0.854
T0 vs. T2				5.845	0.665	227	8.79	<0.001
T1 vs. T2				5.129	0.667	227	7.69	<0.001

Note. Gender was coded as follow: 0 = female; 1 = male; SE = standardized error; df = degrees of freedom; SD = standard deviation; ICC = intraclass coefficient; Det = detachment.

**Table 6:** Linear mixed-effects model with antagonism as moderator.

				Confidence Intervals				
Names	Effect	Estimate	SE	Lower	Upper	df	t	*p*
Fixed coefficients								
Intercept		17.622	0.937	15.779	19.465	15.9	18.810	<0.001
T1	T1–T0	−0.718	0.660	−2.016	0.581	227.2	−1.087	0.278
T2	T2–T0	−5.845	0.658	−7.139	−4.551	227	−8.883	<0.001
Gender		−2.929	1.717	−6.306	0.448	107.1	−1.706	0.091
Age		−0.217	0.276	−0.759	0.325	105.9	−0.787	0.433
Ant		−1.053	1.149	−3.313	1.207	109.7	−0.916	0.362
T1 × Ant	T1–T0	0.173	0.891	−1.579	1.926	227.0	0.195	0.846
T2 × Ant	T2–T0	2.250	0.891	0.498	4.002	227	2.527	0.012
**Random Components**					**Variance**	**SD**	**ICC**	
Participants’ intercepts					67.23	8.20	0.728	
Therapists’ intercepts					3.95	1.99	0.136	
Residual					25.11	5.01		
**Model Fit**					**R^2^**	**df**	**LRT X^2^**	***p***
Conditional					0.765	9	238.044	<0.001
Marginal					0.098	7	89.773	<0.001
**Post-Hoc Comparison: Time**				**Difference**	**SE**	**df**	**T**	***p***
T0 vs. T1				0.718	0.660	227	1.09	0.832
T0 vs. T2				5.846	0.658	227	8.88	<0.001
T1 vs. T2				5.129	0.660	227	7.77	<0.001

Note. Gender was coded as follows: 0 = female; 1 = male; SE = standardized error; df = degrees of freedom; SD = standard deviation; ICC = intraclass coefficient; Ant = antagonism.

**Table 7:** Linear mixed-effects model with disinhibition as moderator.

				Confidence Intervals				
Names	Effect	Estimate	SE	Lower	Upper	df	t	*p*
Fixed coefficients								
Intercept		17.615	0.905	15.834	19.396	15.6	19.457	<0.001
T1	T1–T0	−0.716	0.667	−2.027	0.596	227.2	−1.073	0.284
T2	T2–T0	−5.845	0.665	−7.152	−4.537	227	−8.794	<0.001
Gender		−2.950	1.724	−6.341	0.440	109.0	−1.712	0.090
Age		−0.159	0.269	−0.689	0.371	103.1	−0.590	0.556
Dis		0.680	1.114	−1.511	2.871	96.8	0.610	0.543
T1 × Dis	T1–T0	−0.518	0.886	−2.261	1.226	227.0	−0.584	0.560
T2 × Dis	T2–T0	1.042	0.886	−0.702	2.786	227	1.175	0.241
**Random Components**					**Variance**	**SD**	**ICC**	
Participants’ intercepts					68.80	8.29	0.729	
Therapists’ intercepts					2.19	1.48	0.079	
Residual					25.62	5.06		
**Model Fit**					**R^2^**	**df**	**LRT X^2^**	***p***
Conditional					0.759	9	233.071	<0.001
Marginal					0.092	7	84.838	<0.001
**Post-Hoc Comparison: Time**				**Difference**	**SE**	**df**	**T**	***p***
T0 vs. T1				0.716	0.667	227	1.07	0.852
T0 vs. T2				5.844	0.665	227	8.79	<0.001
T1 vs. T2				5.129	0.667	227	7.69	<0.001

Note. Gender was coded as follows: 0 = female; 1 = male; SE = standardized error; df = degrees of freedom; SD = standard deviation; ICC = intraclass coefficient; Dis = disinhibition.

**Table 8:** Linear mixed-effects model with psychoticism as moderator.

				Confidence Intervals				
Names	Effect	Estimate	SE	Lower	Upper	df	t	*p*
Fixed coefficients								
Intercept		17.6200	0.858	15.932	19.308	12.8	20.5355	<0.001
T1	T1–T0	−0.7143	0.670	−2.031	0.603	227.2	−1.0667	0.287
T2	T2–T0	−5.8448	0.668	−7.158	−4.532	227.0	−8.7558	<0.001
Gender		−2.8502	1.717	−6.227	0.527	110.1	−1.6601	0.100
Age		−0.0163	0.283	−0.574	0.541	89.5	−0.0575	0.954
Psy		1.6480	1.144	−0.603	3.899	88.5	1.4402	0.153
T1 × Psy	T1–T0	−0.3174	0.871	−2.030	1.395	227.1	−0.3645	0.716
T2 × Psy	T2–T0	0.6333	0.869	−1.076	2.343	227.0	0.7286	0.467
**Random Components**					**Variance**	**SD**	**ICC**	
Participants’ intercepts					69.46	8.333	0.729	
Therapists’ intercepts					0.146	0.383	0.006	
Residual					25.85	5.08		
**Model Fit**					**R^2^**	**df**	**LRT X^2^**	***p***
Conditional					0.757	9	232.837	<0.001
Marginal					0.101	7	84.605	<0.001
**Post-Hoc Comparison: Time**				**Difference**	**SE**	**df**	**t**	***p***
T0 vs. T1				0.715	0.670	227	1.07	0.861
T0 vs. T2				5.844	0.668	227	8.75	<0.001
T1 vs. T2				5.129	0.670	227	7.66	<0.001

Note. Gender was coded as follows: 0 = female; 1 = male; SE = standardized error; df = degrees of freedom; SD = standard deviation; ICC = intraclass coefficient; Psy = psychoticism.

## Data Availability

The study was not preregistered. The datasets and the additional materials generated during and/or analyzed during the current study are available from the corresponding author on reasonable request.
